# Myelin Water Imaging Demonstrates Lower Brain Myelination in Children and Adolescents With Poor Reading Ability

**DOI:** 10.3389/fnhum.2020.568395

**Published:** 2020-10-16

**Authors:** Christian Beaulieu, Eugene Yip, Pauline B. Low, Burkhard Mädler, Catherine A. Lebel, Linda Siegel, Alex L. Mackay, Cornelia Laule

**Affiliations:** ^1^Department of Biomedical Engineering, University of Alberta, Edmonton, AB, Canada; ^2^Department of Physics and Astronomy, University of British Columbia, Vancouver, BC, Canada; ^3^Department of Education and Counseling Psychology, University of British Columbia, Vancouver, BC, Canada; ^4^Philips Health Care, Hamburg, Germany; ^5^Department of Radiology, University of Calgary, Calgary, AB, Canada; ^6^Department of Radiology, University of British Columbia, Vancouver, BC, Canada; ^7^Department of Pathology & Laboratory Medicine, University of British Columbia, Vancouver, BC, Canada; ^8^International Collaboration on Repair Discoveries (ICORD), University of British Columbia, Vancouver, BC, Canada

**Keywords:** dyslexia, myelin water, MRI, reading ability, children, adolescent

## Abstract

Magnetic resonance imaging (MRI) provides a means to non-invasively investigate the neurological links with dyslexia, a learning disability that affects one’s ability to read. Most previous brain MRI studies of dyslexia and reading skill have used structural or diffusion imaging to reveal regional brain abnormalities. However, volumetric and diffusion MRI lack specificity in their interpretation at the microstructural level. Myelin is a critical neural component for brain function and plasticity, and as such, deficits in myelin may impact reading ability. MRI can estimate myelin using myelin water fraction (MWF) imaging, which is based on evaluation of the proportion of short T2 myelin-associated water from multi-exponential T2 relaxation analysis, but has not yet been applied to the study of reading or dyslexia. In this study, MWF MRI, intelligence, and reading assessments were acquired in 20 participants aged 10–18 years with a wide range of reading ability to investigate the relationship between reading ability and myelination. Group comparisons showed markedly lower MWF by 16–69% in poor readers relative to good readers in the left and right thalamus, as well as the left posterior limb of the internal capsule, left/right anterior limb of the internal capsule, left/right centrum semiovale, and splenium of the corpus callosum. MWF over the entire group also correlated positively with three different reading scores in the bilateral thalamus as well as white matter, including the splenium of the corpus callosum, left posterior limb of the internal capsule, left anterior limb of the internal capsule, and left centrum semiovale. MWF imaging from T2 relaxation suggests that myelination, particularly in the bilateral thalamus, splenium, and left hemisphere white matter, plays a role in reading abilities. Myelin water imaging thus provides a potentially valuable *in vivo* imaging tool for the study of dyslexia and its remediation.

## Introduction

Dyslexia is a complex learning disability that affects one’s ability to read regardless of intelligence (Siegel, 1989a; Fletcher, 2009). Although the exact cause of dyslexia is still unknown, current hypotheses based primarily on quantitative neuroimaging methods point towards a neurological basis with an emphasis on a left cerebral hemisphere network (Peterson and Pennington, 2012), and in particular the left occipito-temporal cortex (Wandell and Le, 2017; Kronbichler and Kronbichler, 2018). Similarly located anatomical differences have even been seen in pre-readers at risk for dyslexia (Vandermosten et al., 2016). The brain regions implicated include both gray matter (Eckert et al., 2016) and white matter tracts (Wandell and Yeatman, 2013), the latter of which have largely been investigated thus far using diffusion tensor imaging (DTI). Since the earliest DTI papers demonstrating correlations of reading ability with DTI parameters (Klingberg et al., 2000; Nagy et al., 2004; Beaulieu et al., 2005; Deutsch et al., 2005; Niogi and McCandliss, 2006), a vast literature over the last 15 years has highlighted key white matter connections presumed to underlie the potentially inefficient communication or “disconnection” between brain regions in dyslexia (see Ben-Shachar et al., 2007; Vandermosten et al., 2012b; for comprehensive reviews and a meta-analysis of earlier DTI papers). These DTI reading findings are not limited to English with similar results across multiple languages (Qiu et al., 2008; Steinbrink et al., 2008; Thiebaut de Schotten et al., 2014; Zhang et al., 2014; Cui et al., 2016; Takeuchi et al., 2016; Zhao et al., 2016; de Moura et al., 2016; Vanderauwera et al., 2017; Su et al., 2018; Žarić et al., 2018; Moulton et al., 2019). While many of these studies reported DTI changes in the left temporo-parietal white matter, other bilateral regions and the corpus callosum have also shown associations with reading, implicating a broader network.

Regional white matter DTI has been correlated to language ability and pre-reading skills in younger “pre-readers” (Saygin et al., 2013; Vandermosten et al., 2015; Wang et al., 2017; Dodson et al., 2018; Vanderauwera et al., 2018; Walton et al., 2018; Ozernov-Palchik et al., 2019; Hutton et al., 2020), and even in infants with a family history of developmental dyslexia (Langer et al., 2017). Longitudinal DTI has shown that both baseline values and changes of diffusion parameters between scans predict future reading ability in healthy controls (Hoeft et al., 2011; Yeatman et al., 2012; Myers et al., 2014; Gullick and Booth, 2015; Takeuchi et al., 2016; Vanderauwera et al., 2017; Borchers et al., 2019; Bruckert et al., 2019; Lebel et al., 2019) and in neurodevelopmental disorders such as fetal alcohol spectrum disorders (Treit et al., 2013). Further compelling evidence of a link between regional white matter plasticity and function comes from DTI parameter changes with remediation in grade-school poor readers (Keller and Just, 2009; Huber et al., 2018), training in spelling-impaired children (Gebauer et al., 2012), and acquisition of literacy in those learning to read in adulthood (Thiebaut de Schotten et al., 2014). Most DTI studies of reading focus on the white matter and do not analyze the deep gray matter structures, but DTI has shown correlations in the left and right thalamus with reading ability in adolescents and young adults (Lebel et al., 2013).

Although DTI has implicated certain white matter regions and connections for adequate reading, the interpretation of what is underpinning these findings at the microstructural level is not specific. Myelin is a major component of white matter and, given its function of optimizing impulse transmission, may be important in performing complex, “multi-cortex” tasks efficiently, as well as promoting plasticity during learning (Zatorre et al., 2012). Water diffusion parameters are not specific to myelin (Beaulieu and Allen, 1994) and can be affected by gross anatomical changes as well as myelin content, axon diameter, axon packing density, extra-axonal space, and dispersion of fiber orientations within an imaging voxel (for review see Beaulieu, 2002). More specific microstructural interpretation may come from the diffusion coefficients relative to the length of the axons including radial diffusivity (RD), where increases are thought to reflect demyelination (Song et al., 2002, 2003). Reading intervention studies have shown reductions of RD over time, interpreted as increases in myelin (Keller and Just, 2009; Gebauer et al., 2012); however, RD from the DTI model is inadequate in regions of crossing fibers (Wheeler-Kingshott and Cercignani, 2009) and may only be specific after a known timed injury to a tract (Concha et al., 2006). Although more comprehensive models of diffusion magnetic resonance imaging (MRI) data have been applied in several reading studies to reveal better tracking of white matter tracts at crossing regions and correlations of new diffusion model parameters (e.g., HMOA, hindrance modulated orientational anisotropy; AWF, axonal water fraction; ICVF, intracellular volume fraction) with reading ability (Vanderauwera et al., 2015; Zhao et al., 2016; Huber et al., 2019), these advanced diffusion MRI models are not specific to myelin.

A few alternative imaging strategies beyond diffusion MRI have been applied to reading and language studies to potentially enhance specificity to microstructure. Myelin content in brain regions has been inferred or estimated primarily *via* water (hydrogen) relaxation properties in the tissue. Myelin water fraction (MWF), derived from an MRI technique called mcDESPOT (multicomponent driven equilibrium single pulse observation of T1 and T2), and its hemispheric asymmetry has been positively correlated with receptive and expressive language ability in typically developing infants and children under 6 years of age (O’Muircheartaigh et al., 2013, 2014). The left arcuate fasciculus showed higher T1-weighted image intensity (by ~1%) in preliterate children at risk for dyslexia (Kraft et al., 2016); however, this is not a quantitative measure of relaxation. The absolute longitudinal relaxation time (T1 in ms) was lower in “reading” tracts relative to “math” tracts, which the authors interpreted as greater myelination in the former (Grotheer et al., 2019), although T1 is also non-specific and there was no assessment of correlations of T1 with reading ability in their typical adult cohort.

A promising myelin-specific imaging option is multi-exponential T2 relaxation based on the premise that a multi-echo T2 decay curve from the brain can be fit to different components (see reviews by MacKay and Laule, 2016; Does, 2018). The shortest T2 component arises from water trapped between myelin bilayers, and the ratio of the short T2 component signal to the total signal yields the MWF per voxel. This short T2 component appears to be specific to myelin, as validated by histology in excised nerve samples (Fenrich, 1992; Does and Snyder, 1996; Beaulieu et al., 1998; Webb et al., 2003) as well as brain and spinal cord tissue from multiple sclerosis patients (Laule et al., 2006, 2008, 2016). While the T2-relaxation based MWF method has been used to investigate normal development and many diseases (MacKay and Laule, 2016), to our knowledge, it has not been used to study reading in children. We aimed to examine the relationship between reading ability and regional brain myelin content determined by multi-exponential T2 in children and adolescents with a wide range of reading abilities.

## Materials and Methods

### Participants

A total of 20 participants (four females, 16 males; a range of 10–18 years with 15/20 between 10–13 years; median = 12.2 years) with diverse reading ability were recruited from several school districts in the Greater Vancouver Regional District, British Columbia. The participants were carefully selected from either a prior non-MRI longitudinal reading study where they were assessed yearly from kindergarten to grade 4 (Lipka et al., 2006) or from students who attended a specialized school for learning disabilities. Individuals identified with poor reading scores over multiple years in the longitudinal study or those identified as having a reading disability from the school were invited, as well as good readers from the longitudinal study. Informed written consent, as approved by the Clinical Research Ethics Board, was obtained for all participants.

### Cognitive and Reading Assessments

The assessments were performed by two trained and experienced individuals (coauthor PL and a research assistant) overseen by a reading specialist (coauthor LS). The Woodcock-Johnson-III (WJ-III) test of cognitive abilities was used for a brief assessment of intelligence that includes three components: verbal comprehension (a test of vocabulary and analogies), concept formation (a test of fluid reasoning), and visual matching (a test of processing speed). The results of the three components were combined to give a Brief Intelligence Ability (BIA) score that provided a measurement of intelligence in approximately 10–15 min. Reading ability was measured using three reading assessments: (1) the Reading Subset of the Wide Range Achievement Test (WRAT3 Reading)—a test of recognition and naming of letters as well as pronunciation of words with increasing complexity and unfamiliarity; (2) WJ-III Letter-Word Identification (Word ID)—a test of the ability to name letters and words from a list; and (3) WJ-III Word Attack—a test of phonetic skills by pronouncing nonsense words quickly. All raw scores were converted to age-normalized standard scores and population percentiles relative to the test’s published norms.

Concerning the classification of participants as either good or poor readers, the IQ-reading discrepancy definition was not used here because of the overwhelming evidence of its lack of validity (see e.g., Siegel, 1989b; Fletcher et al., 1992; Tanaka et al., 2011; Siegel and Hurford, 2019). Specifically, children were classified as poor readers (dyslexia) if at least two of their reading assessments were below the 25th percentile, while good readers had all reading scores above the 35th percentile. If participants did not meet this poor reader criterion but had at least one score below the 35th percentile, they were classified as intermediate readers. Intermediate readers were included in the correlation analysis, but not the group comparisons. The 25th percentile cut-off has been used previously to define children with a reading disability in group comparisons (Siegel, 1989a; Stanovich and Siegel, 1994).

### MRI Acquisition

**Figure 1:**
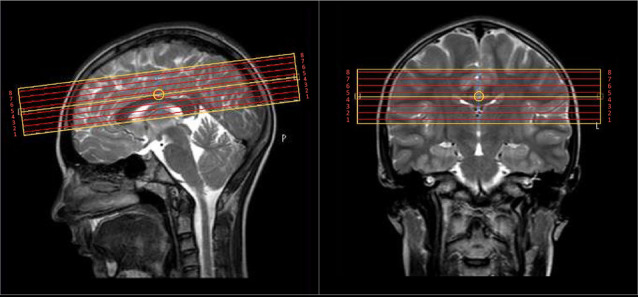
The 4 cm coverage over eight slices of the multi-echo T2 scan was centered on the body of the corpus callosum.

Brain MRI was acquired on a 3T Philips Achieva scanner. T2 relaxation was measured with a 32-echo 3D GRASE (Prasloski et al., 2012b) with TR = 1,500 ms, TE = 10 ms, echo spacing 10 ms, two gradient echoes per spin echo, 8 transverse slices, voxel size = 1 × 1.8 × 5 mm^3^, flip angle = 90°, field of view = 240 × 180 × 40 mm^3^, scan time = 11:39 min. The inferior/superior coverage was limited by the MRI scanner technology at the time of data collection [circa 2006—(Yip et al., 2010)] and considerations for keeping the scan time minimized for our children/adolescent cohort. The 3D slab was centered on the corpus callosum to provide sufficient and consistent coverage over all participants for many-core white and deep gray matter regions of interest (Figure 1). To assist in the identification of structures for region-of-interest (ROI) analysis, a high resolution 3D T1-weighted Turbo Field Echo was also acquired with TR = 10 ms, TE = 6 ms, TI = 848 ms, 120 axial slices, voxel size = 1.1 × 1.1 × 1.1 mm^3^, flip angle = 80°, TFE factor = 154, sense factor 1.7, field of view = 212 × 181 × 132 mm^3^, scan time = 5:02 min.

### MRI Analysis

The raw 3D GRASE multi-echo images were inspected to confirm the lack of motion artifacts. T2 distributions were calculated for every voxel in the T2 relaxation decay data set using a regularized non-negative least squares (NNLS) algorithm (Whittall and MacKay, 1989) accounting for stimulated echo artifacts due to radiofrequency (B1+), as previously described (Prasloski et al., 2012a). MWF was defined by the area under the T2 distribution below 40 ms divided by the total area to yield voxel-wise MWF maps.

Multi-echo T2 images were registered to the T1 images using FSL (Smith et al., 2004), the latter used for manual ROI placement by one individual (coauthor EY) of 10 white matter and six gray matter structures including the genu and splenium of the corpus callosum, as well as bilateral (left and right kept separate) posterior limb of the internal capsule, anterior limb of the internal capsule, minor forceps, centrum semiovale, thalamus, putamen, and caudate nucleus. These ROIs were selected as they are readily identifiable in the acquired slab and cover commissural, projection, and association white matter regions, as well as core deep gray matter regions, some of which have been implicated in prior imaging studies of dyslexia/reading. For example, the centrum semiovale has been shown in numerous previous DTI studies to have a link to reading test performance (e.g., see Figure 2 in Ben-Shachar et al., 2007).

**Figure 2:**
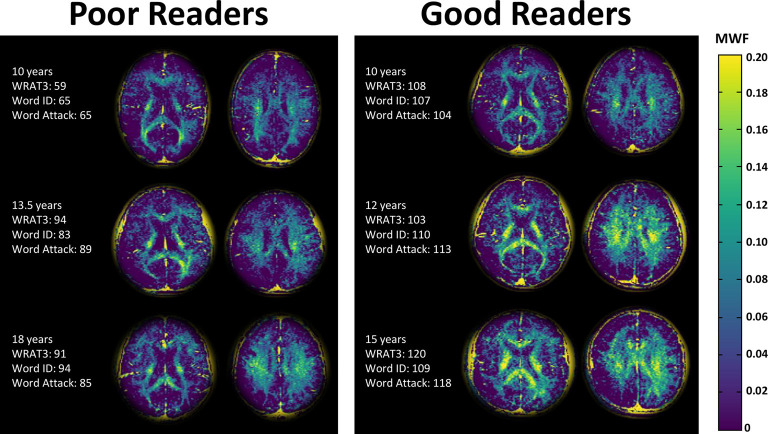
Myelin water fraction (MWF) maps of two slices in three poor readers and three good readers of similar ages. The MWF maps highlight the white matter, as expected, but shows noticeably higher MWF in the good readers. Note that the background has been cropped and the residual yellow skull is an artifact of the processing.

### MWF Group Comparison and Correlation Analysis

Age, intelligence scores, and reading scores were compared between good and poor readers with a two-tailed Student’s test. A one-tailed Student’s *t*-test was performed for the group comparison to determine if MWF was lower in poor readers relative to good readers. Pearson’s correlation coefficients assessed linear correlations between age, intelligence (WJIII BIA Brief IQ), and the three age-standardized reading scores (WRAT-3 Reading, WJ-III Word ID, WJ-III Word Attack) with MWF within each of the 16 ROIs over the entire group. Note that age was not a covariate for the MWF assessments since MWF did not correlate with age (see “Results” section). A Benjamini Hochberg false discovery rate (FDR) correction of 0.05 was applied to account for multiple comparisons (uncorrected *p-values* surviving FDR correction are reported).

## Results

### Demographics

Of the 20 participants, seven were classified as poor readers (one female, six males), 11 as good readers (three females, eight males), and two as intermediate readers (two males). There were marked group differences in all three age-normalized reading tests with the good readers yielding much higher scores (mean 107–112) than the poor readers (mean 84–89; Table 1). The handedness was right in 18 participants and left in two participants (both good readers). There were no significant differences in age (mean ~12.8 years) or intelligence scores between good and poor readers, and no significant correlations between age and any age-standardized cognitive or reading assessment.

**Table 1:** Subject demographics with age-standardized scores showing similar age and intelligence, but significantly different reading scores between the good and poor reading groups [mean (SD)].

	Good readers (*n* = 11)	Poor readers (*n* = 7)	*p*-value
Age (years)	12.8 (1.6)	12.8 (2.8)	0.93
WJ III BIA (Brief IQ)^a^	100 (15)	94 (16)	0.36
Verbal comprehension	108 (12)	101 (13)	0.23
Concept formation	113 (16)	107 (18)	0.44
Visual matching	84 (14)	81 (12)	0.71
WRAT3 reading	112 (11)	84 (12)	0.0001*
WJ III letter-word Identification	111 (10)	89 (15)	0.001*
WJ III word attack	107 (8)	88 (3)	0.00001*

^a^The Brief Intelligence Assessment (BIA) for IQ is the combination of the subsequent three tests on verbal comprehension, concept formation, and visual matching. *Statistically significant difference between good and poor readers (*p* < 0.001). Note: two other participants (both 11-year-olds) were considered intermediate readers.

### Group Comparisons of Myelin Water Fraction Between Good and Poor Readers

**Figure 3:**
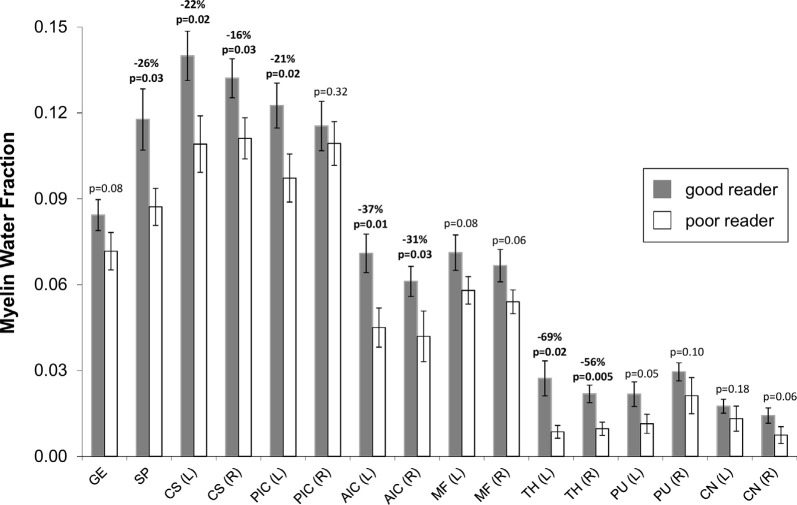
Mean (± standard error) MWF for good (dark bars, *n* = 11) and poor (light bars, *n* = 7) readers for various bilateral (L, left; R, right) white and gray matter structures: genu of corpus callosum (GE), splenium of corpus callosum (SP), posterior limb of the internal capsule (PIC), anterior limb of the internal capsule (AIC), minor forceps (MF), thalamus (TH), putamen (PU) and head of caudate nucleus (CN). MWF was significantly lower (*p* < 0.05) in 8 of 16 regions and showed trend level reduction (*p* = 0.05–0.08) in another five regions for poor readers. As expected, the MWF was higher in the white matter regions (0.06–0.14 for good readers, 0.04–0.11 for poor readers) relative to deep gray matter (0.015–0.03 for good readers, 0.005–0.02 for poor readers).

The MWF maps showed the expected greater MWF in white matter than cortical or deep gray matter, as well as regional variation across the white matter (Figures 2, 3). Notably, the MWF map intensity was visually lower in the poor readers relative to the good readers (see Figure 2 for three examples of each group). MWF was significantly lower in the poor reader group relative to the good reader group in the left and right thalamus by 69% (*p* = 0.02) and 56% (*p* = 0.005), respectively, and in six white matter regions including left (−37%, *p* = 0.01) and right (−31%, *p* = 0.03) anterior limb of internal capsule, left (−22%, *p* = 0.02) and right (−16%, *p* = 0.03) centrum semiovale, left posterior limb of internal capsule (−21%, *p* = 0.02), and splenium of corpus callosum (−26%, *p* = 0.03; Figure 3).

**Figure 4:**
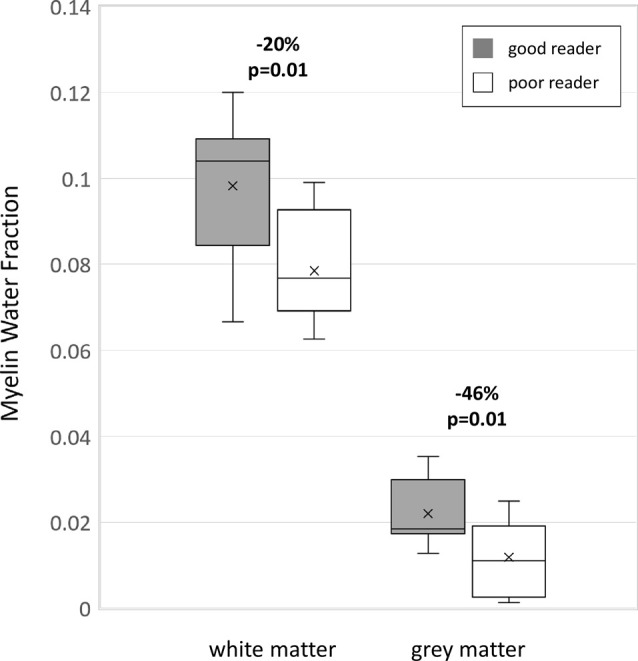
Boxplots of MWF for good (*n* = 11) and poor (*n* = 7) readers over all 10 white matter regions combined and all six deep gray matter regions combined showing marked MWF differences (horizontal line, median; x, mean; box lower edge, first quartile; box upper edge, third quartile; error bars, minimum and maximum data values).

When all 10 white matter ROIs were averaged together (left and right combined) per individual, the poor reader group had 20% less MWF compared to the good reader group (MWF mean ± SE: poor 0.079 ± 0.004 vs. good 0.098 ± 0.004, *p* = 0.011; Figure 4). Similarly, the mean of the six deep gray matter ROIs also showed that MWF was lower by 46% in the poor reader group compared to the good reader group (MWF poor 0.012 ± 0.002 vs. good 0.022 ± 0.002, *p* = 0.011; Figure 4).

### Correlation Analysis of Myelin Water Fraction With Reading

**Figure 5:**
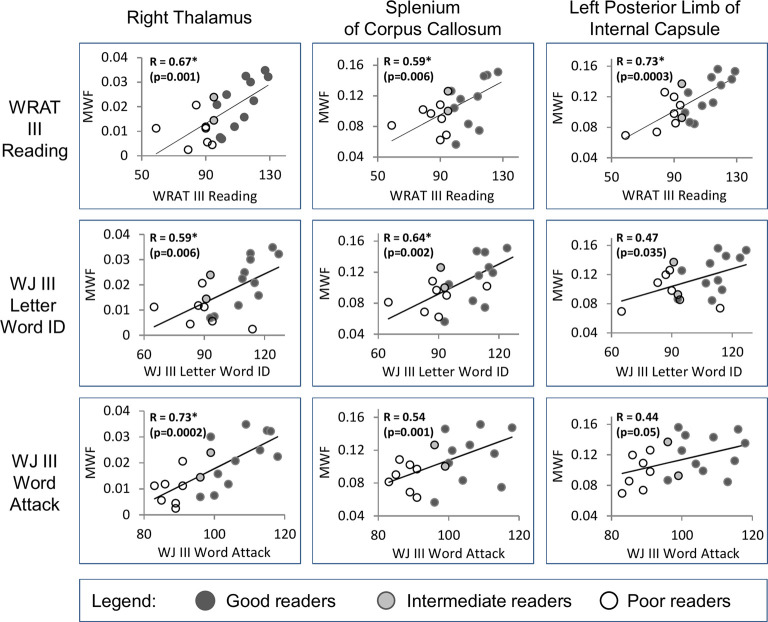
Examples are shown from three regions (right thalamus, splenium of the corpus callosum, and left posterior limb of internal capsule) that show significant positive correlations [*false discovery rate (FDR) 0.05, uncorrected p shown] between MWF and the three reading assessment scores (WRAT III Reading, WJ III Letter Word ID, WJ III Word Attack) in the full group of 20 individuals. The reader groups are shown with different colored symbols.

There were 10 significant (FDR 0.05) positive correlations (*R* = 0.59–0.73) of MWF with the age-standardized reading scores (WRAT3 Reading; WJIII Word ID, Word Attack) over 6 ROIs including the right thalamus with correlations to all three reading scores, left thalamus and splenium of the corpus callosum with two correlations each, and then three left white matter regions with one correlation each (the left posterior limb of the internal capsule, left anterior limb of the internal capsule, left centrum semiovale)—see Table 2 and example plots for three structures in Figure 5. All 6 of these ROIs showed MWF correlations to the WRAT3 Reading score whereas WJ III Word ID was limited to three regions (bilateral thalamus and splenium) and WJ III Word Attack to only the right thalamus. There were no significant correlations between MWF and age (mean *R* = −0.01, mean *p* = 0.67; data not shown) or WJIII BIA Brief IQ (mean *R* = 0.22, mean *p* = 0.63; data not shown). Note that adding age as a covariate to the linear regression yielded comparable R values of 0.62–0.75 for these same 10 correlations (data not shown). Notably, MWF was positively correlated at *p* ≤ 0.05 (not FDR corrected) with age-standardized reading scores for 23/48 correlations performed covering 11/16 ROIs (Table 2).

**Table 2:** Linear Pearson correlations (R/uncorrected p) between three reading assessment scores and myelin water fraction from 16 bilateral (L—left and R—right) white and deep gray matter structures in the overall cohort of 20 participants.

MWF Region	WRAT3 Reading	WJ III Letter-Word ID	WJ III Word Attack
White Matter—R (p)
Genu corpus callosum	0.21 (0.38)	−0.07 (0.77)	0.36 (0.12)
Splenium corpus callosum	0.59 (0.006)*	0.64 (0.002)*	0.54 (0.01)
Centrum semiovale (L)	0.64 (0.003)*	0.42 (0.07)	0.52 (0.02)
Centrum semiovale (R)	0.50 (0.03)	0.41 (0.07)	0.51 (0.02)
Posterior limb internal capsule (L)	0.73 (0.0003)*	0.47 (0.03)	0.44 (0.05)
Posterior limb internal capsule (R)	0.32 (0.18)	0.23 (0.32)	0.26 (0.27)
Anterior limb internal capsule (L)	0.64 (0.002)*	0.42 (0.06)	0.54 (0.01)
Anterior limb internal capsule (R)	0.49 (0.03)	0.25 (0.29)	0.53 (0.02)
Minor forceps (L)	0.49 (0.03)	0.35 (0.14)	0.18 (0.45)
Minor forceps (R)	0.13 (0.59)	0.04 (0.85)	0.11 (0.65)
Deep Gray Matter—R (p)
Thalamus (L)	0.71 (0.0004)*	0.61 (0.004)*	0.54 (0.01)
Thalamus (R)	0.67 (0.001)*	0.59 (0.006)*	0.73 (0.0002)*
Putamen (L)	0.56 (0.01)	0.31 (0.18)	0.27 (0.26)
Putamen (R)	0.44 (0.05)	0.21 (0.37)	0.39 (0.09)
Caudate nucleus (L)	0.32 (0.17)	0.06 (0.79)	0.19 (0.42)
Caudate nucleus (R)	0.33 (0.16)	0.13 (0.58)	0.43 (0.06)

*Indicates significant with FDR 0.05.

## Discussion

Multicomponent T2 relaxation measurements have demonstrated that on average MWF is 20% lower in white matter and 46% lower in deep gray matter ROIs for children who are poor readers compared to good readers. Of all regions, the group comparison highlighted the left and right thalamus with the most marked MWF reductions of 69% and 56% in poor readers. These proportional differences in MWF, which have not been measured before in reading studies, are much greater than those reported for DTI parameters or volumes. MWF is a completely different and more specific tissue microstructure measurement, and such notable differences in proportional changes between imaging measurements are not incongruent. These preliminary results are encouraging in a small, highly selected, and well-characterized sample, and provide the impetus for confirmation in a larger independent population using faster MWF methods with greater brain coverage. The positive correlation of MWF with three different age-standardized scores of reading ability over the entire cohort of 20 readers also implicated the bilateral thalamus, as well as white matter including the splenium of the corpus callosum and left hemisphere regions of the anterior and posterior limb of the internal capsule and more superior in the centrum semiovale, which are consistent with the locations identified in many previous DTI reports of reading ability (reviewed in Vandermosten et al., 2012b). MWF was not correlated with intelligence supporting the notion that dyslexia is a condition that affects reading ability independently of intelligence (Siegel, 1989a; Fletcher, 2009). MWF was also not correlated with age over our 10–18-year-old group, but this finding should be interpreted with caution as 75% of the children were in a narrow range of 10–13 years and the only individual older than 15 years was a poor reader. Other myelin-sensitive MR methods have reported changes across a similar age span. For example, the volume fraction of myelin derived by mcDESPOT has been shown to increase in the white matter of 50 typically developing children between 6–15 years (Geeraert et al., 2019), although they used a different myelin content imaging method than our study, with a larger sample that included younger participants, but did not include poor readers.

### Anatomy of Reading Circuitry

The areas seen here have been previously highlighted by neuroimaging studies of brain structure and reading. It has been suggested that learning to read is mediated by white matter connections through the splenium of the corpus callosum (Carreiras et al., 2009). Left lateralization of white matter tracts is commonly observed showing links to genetic factors and age-related changes in the perisylvian language networks (Budisavljevic et al., 2015) and links to phonological processing with the arcuate fasciculus in children (Lebel and Beaulieu, 2009). Additional evidence for a direct role of white matter in reading comes from a neurosurgical case report of subcortical stimulation of a specific anterior/posterior tract connecting the parietal and frontal lobes in the left hemisphere (Motomura et al., 2014). Another case report highlighted the inability to read or identify letters after a biopsy of a left thalamic mass that also induced damage to the splenium of the corpus callosum (Tamhankar et al., 2004); our results suggest that both these structures play a role in reading. Left lateralized white matter connections, and the splenium of the corpus callosum through its role in vision, are core parts of the reading circuitry (Wandell and Le, 2017). Although the left hemisphere has been a primary focus of many studies, brain regions in the right hemisphere have also been implicated in DTI (for example Hoeft et al., 2011; Gebauer et al., 2012; Vandermosten et al., 2012b; Lebel et al., 2013) and fMRI (Maisog et al., 2008) studies, suggesting a more distributed widespread network for reading skill, which is also in-line with our observations showing reduced myelin in several right-sided brain regions.

### The Role of Myelin in Reading Circuitry and Plasticity

The microstructural underpinnings which contribute to abnormalities in this circuitry are not confirmed, but it is conceivable that myelination is an important factor. As the primary role of myelin is to increase conduction speed along axons, poor myelination of key brain regions involved in reading may contribute to dyslexia. This theory is consistent with several studies that have shown that individuals with dyslexia manifest rate processing problems (Cohen-Mimran and Sapir, 2007; Murphy and Schochat, 2009; Wright and Conlon, 2009). Myelination is also important for white matter plasticity which is critical for brain circuit formation and function (Zatorre et al., 2012; Chorghay et al., 2018). Longitudinal DTI studies of remediation in dyslexia have shown post-intervention reductions of RD in the left anterior centrum semiovale (Keller and Just, 2009) as well as the left/right corona radiata and posterior limb of the internal capsule (Gebauer et al., 2012), which may be consistent with increased myelination (Song et al., 2002, 2003). Other DTI studies reporting lower RD with better reading scores have also implicated that myelination is driving the white matter fractional anisotropy differences in the reading disabled cohorts in white matter regions such as left arcuate fasciculus (Vandermosten et al., 2012a; Thiebaut de Schotten et al., 2014; Christodoulou et al., 2017; Hutton et al., 2020), anterior and superior corona radiata (Frye et al., 2011), and left anterior limb of the internal capsule (Qiu et al., 2008). Notably, these regions are all consistent with our findings. The typical trajectory of RD reduction with age over 6–16 years observed in controls was not present in children with dyslexia in the inferior frontal occipital and posterior limb of the internal capsule (Rollins et al., 2009).

### MWF and Cognitive Measures

This study contributes to a fairly sparse literature on how MWF relates to cognitive measures. A small cross-sectional study in preadolescent males aged 9–12 years found a strong correlation between MWF and verbal IQ (Whitaker et al., 2008). Experience-dependent changes in myelin due to motor training have been shown with multi-exponential T2 relaxation derived MWF, suggesting that interventions can change MWF (Lakhani et al., 2016). mcDESPOT is a methodologically different approach to estimating myelin content which makes use of T1-weighted and steady-state gradient-echo acquisitions to estimate T1 and T2 times of the tissue components in the brain (Deoni et al., 2008). mcDESPOT has yielded measures related to myelin content that correlate with performance in language tasks, in particular, hemispheric asymmetry positively correlating with receptive and expressive language ability in typically developing infants and children under 6 years of age (O’Muircheartaigh et al., 2013, 2014). mcDESPOT derived myelination measures in the splenium of the corpus callosum also correlated positively with Early Learning Composite score (sum of fine motor, visual reception and expressive and receptive language) in children less than 5 years old, with more widespread bilateral white matter involvement if only examining 1–2-year-old toddlers (Deoni et al., 2016), further supporting a link between enhanced myelination in white matter and cognitive function. However, it should be noted that the spatial patterns of T2-based and mcDESPOT-based myelin measures are quite different in the human brain (Zhang et al., 2015a,b).

### Deep Gray Matter and Reading Ability

Our results suggest that myelination of brain circuitry involved in reading ability is not limited to white matter. The bilateral thalamus MWF in poor readers was much lower (69%% left and 56% right) than good readers (MWF poor ~0.01 vs. good 0.02–0.025). It is important to keep in mind that the absolute MWF in deep gray matter is much smaller than in white matter. The right thalamus MWF yielded strong positive correlations independently with all three reading scores (*R* = 0.59, 0.67, 0.73), while the left thalamus MWF correlated with two reading scores (*R* = 0.61, 0.71). These observations are in line with the possible functional consequences of myelin abnormalities within the thalamus, which is critical for cognitive functioning and language (Llano, 2013; Sherman, 2016), and acts as a “relay station” for connections between various brain regions. The different thalamic subparts are separated by myelinated lamellae, and less myelin could lead to deficits in information integration and relay. Post-mortem studies of dyslexia have shown cytoarchitecture abnormalities of neurons within the thalamus including the lateral geniculate nucleus (LGN), the primary processing center for visual information located inside the thalamus (Wandell and Le, 2017), and the left medial geniculate nucleus (MGN), a portion of the auditory thalamus hypothesized to influence attention (Galaburda et al., 1994).

Other MRI studies have also reported several thalamic abnormalities related to dyslexia and reading ability. Individuals with dyslexia have smaller and differently shaped LGN (Giraldo-Chica et al., 2015). Fractional anisotropy in the left and right thalamus has correlated with reading ability (Lebel et al., 2013) and diffusion tensor tractography has identified differences in thalamic connections in individuals with dyslexia (Fan et al., 2014; Müller-Axt et al., 2017; Žarić et al., 2018; Tschentscher et al., 2019). These studies align with our observations of lower MWF in the anterior limb of the internal capsule which contains axons connecting the thalamus and frontal lobe. Functional MRI activity of the thalamus (including right side) also differs in people with dyslexia (Maisog et al., 2008; Díaz et al., 2012; Paz-Alonso et al., 2018) and may be altered following reading intervention (Barquero et al., 2014). These functional changes could be partly a result of changes in myelination, but fMRI was not acquired in our study for comparison to MWF.

### Limitations and Future Directions

A clear limitation of our study is the small sample size of 20 participants, although they were a carefully selected sample from a previous longitudinal reading study from kindergarten to Grade 4 and were identified with a reading disability from a specialized school for learning disabilities as supervised by a long time expert in dyslexia (coauthor LS; Siegel, 1989a; Stanovich and Siegel, 1994; Lipka et al., 2006). Further, if the large MWF changes are confirmed in future studies, very large sample sizes may not be required if reading disabled participants are selected and characterized appropriately. The influence of sex and handedness was not possible to evaluate as there were only two left-handers (both good readers) and only four females where one was classified as a poor reader vs. six males. The MWF-reading linear correlations could be driven by marked group differences of MWF between good and poor readers, although such an MWF-reading relationship appears to exist within each group (e.g., right thalamus MWF vs. WRAT III in good readers; Figure 5), granted such an assessment is beyond the scope of our small sample size. Our pilot study provides compelling evidence for larger replication studies of myelin in reading and dyslexia using faster MWF imaging techniques yielding more extensive data collection over the whole brain (Alonso-Ortiz et al., 2015; Lee et al., 2020). That said, our average white matter MWF was 0.10 in the good readers, which fits with a very recent report of 0.10–0.11 in adults using the same type of MRI pulse sequence (Ocklenburg et al., 2019). Our study focused on manual ROI measurements in nine specific regions that were identifiable in all participants within the 4 cm slab and covered various types of white matter as well as the deep gray matter relevant to the reading literature. With whole-brain MWF coverage and the acquisition of coregistered diffusion imaging, future work could include tract specific analysis methods with tractography to extract MWF along tracts of interest in reading, e.g., the arcuate fasciculus (Baumeister et al., 2020). Like many quantitative MRI techniques, the assignment of the short T2 component to “myelin water fraction” is not proof that there are myelin differences in the dyslexic brain, but there is much histological evidence supporting this hypothesis, as outlined in the Introduction. However, the presence of iron can shorten the water T2 in tissue and could interfere with the correspondence of the short T2 component to myelin specifically (Birkl et al., 2019). Although iron is typically far more concentrated in deep gray matter structures, it is also present in the oligodendrocytes, whose cell processes wrap the myelin sheaths of white matter. Hence an alternative interpretation of our results could, in part, be reduced iron (fewer oligodendrocytes) in poor readers, which could be evaluated using quantitative susceptibility mapping MRI.

In conclusion, multi-exponential T2 relaxation can help elucidate the microstructural brain differences associated with the acquisition and performance of cognitive abilities such as reading. This study suggests that a defect in myelination could underlie inefficient communication between brain regions in individuals who are poor readers. Myelin water imaging is complementary to other quantitative MRI techniques and is a potentially valuable *in vivo* imaging tool for the study of dyslexia and its remediation.

## Data Availability Statement

The raw data supporting the conclusions of this article will be made available by the authors, without undue reservation.

## Ethics Statement

The studies involving human participants were reviewed and approved by Clinical Research Ethics Board, University of British Columbia. Written informed consent to participate in this study was provided by the participants’ legal guardian/next of kin.

## Author Contributions

All authors contributed to writing the manuscript, interpreting the data, and approved the submitted version. Also, CB: designed the study and secured the funding. EY: analyzed the data. PL: acquired the data. BM: designed the study and acquired the data. CAL: designed the study. LS: designed the study, acquired the data, and secured the funding. AM: designed the study and acquired the data. CL: analyzed the data.

## Conflict of Interest

BM was employed by the company Philips Healthcare.

The remaining authors declare that the research was conducted in the absence of any commercial or financial relationships that could be construed as a potential conflict of interest.
